# Ubiquinol Effect on Sperm Parameters in Subfertile Men Who Have Astheno-Teratozoospermia With Normal Sperm Concentration

**DOI:** 10.5812/numonthly.16870

**Published:** 2014-05-10

**Authors:** Basri Cakiroglu, Seyit Erkan Eyyupoglu, Ramazan Gozukucuk, Bekir Sami Uyanik

**Affiliations:** 1 Department of Urology, Hisar Intercontinental Hospital, Istanbul, Turkey; 2 Department of Urology, Sabuncuoglu Serefeddin Training and Research Hospital, Amasya, Turkey; 3 Department of Infectious Disease and Clinical Microbiology, Hisar Intercontinental Hospital, Istanbul, Turkey; 4 Department of Clinical Biochemistry, Hisar Intercontinental Hospital, Istanbul, Turkey

**Keywords:** Infertility, Oligospermia, Idiopathic, Ubiquinol

## Abstract

**Background:**

Considering all the couples willing and trying to get pregnant, the incidence of infertility is 15% of which approximately half of the cases are due to the male factors.

**Objectives:**

The aim of this study was the investigation of the effects of ubiquinol, reduced form of coenzyme Q10 (Co-Q10), an empiric treatment modality, on sperm parameters in idiopathic subfertility.

**Patients and Methods:**

In this retrospective study, 62 patients who had received 100 mg ubiquinol twice a day for six months due to idiopathic infertility since January 2012 to January 2013 were included. Only infertile patients with astheno-teratozoospermia without any identified etiology and with a spermatozoa concentration of greater than 13 × 10^6^/mL were included.

**Results:**

The increase in mean values of concentration after the ubiquinol treatment was not statistically significant (P value = 0.065). However, the changes in morphology and motility (fast progressive [*a*] and *a* + slow progressive [*b*]) were statistically significant (P < 0.00).

**Conclusions:**

The weakness of the literature with regard to coenzyme Q10 is about its effects in patients with severely diminished sperm densities and the physiologic steps of morphologic improvements.

## 1. Background

**Figure 1.**
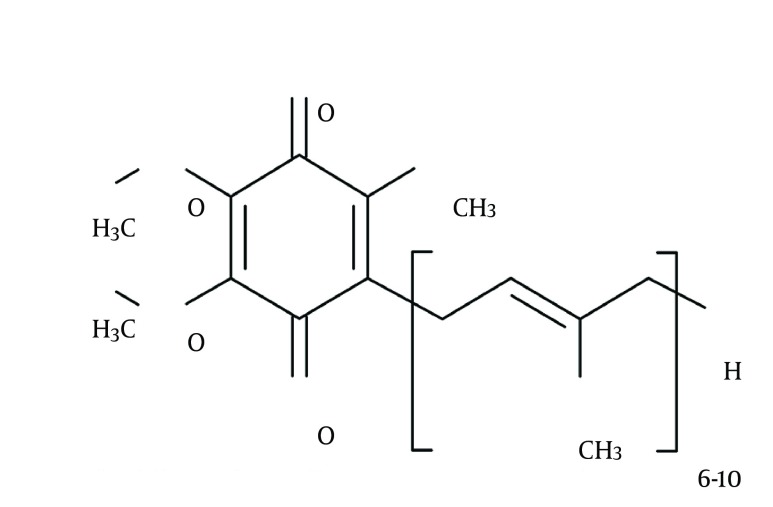
The Chemical Structure of Coenzymes Q10

Considering all the couples willing and trying to conceive, the incidence of infertility is 15% of which approximately half of the cases are due to the male factors (1). Multiple factors including hormonal or genetic disorders, previous infections, previous genital, gonadal, or retroperitoneal surgeries, autoimmune factors, systemic diseases, heavy metals intoxication, smoking, gonadotoxic agents, radiation, side effects of drugs, and postponing or smoothness materials play a role in male infertility; however, varicocele is the most common finding amongst males with primary infertility and fortunately, it is a treatable disease (2). Despite performing all necessary investigations, in more than 25% of all cases with male infertility the underlying etiology could not be found (3). During the last 15 years, the overproduction of free radicals or reactive oxygen species (ROS) has been studied largely as a cause of sperm destruction and it has been concluded as a mechanism of infertility (4). Some of the ROS examples in seminal plasma are superoxide anion, hydroxyl radicals, and hydrogen peroxide. Although oxygen is indispensable for life, these oxygen derivatives alter cellular functions and threaten the cellular life (3). Consequently, in order to carry on the cellular functions normally, ROSs should be inactivated continuously. This inactivation process is performed by antioxidants present in seminal plasma. In addition to the enzymatic antioxidants including superoxide dismutase, catalase, and glutathione peroxidase, the existence of the nonenzymatic antioxidant molecules such as vitamin C, vitamin E, pyruvate, glutathione, acetyl cysteine, carotene, coenzyme Q10 (Co-Q10 [ Figure 1 ]), and carnitine are determined in semen (5). The production of ROS in slight amounts is necessary for capacitation, which is a physiological function. On the other hand, creation of ROS in an amount beyond the total antioxidant capacity (TAC) is harmful for spermatozoa and the difference in favor of ROS in this balance determines the oxidative stress. Spermatozoal oxidative stress (SOS), while damages sperm membrane permeability by the way of lipid peroxidation, results in formation of impaired DNA due to degradation, fragmentation, and cross-linking proteins (6). It has been postulated that owing to an increase in antioxidant enzyme capacity with the additional Co-Q10 support, a positive effect on TAC and a decrease in SOS levels may be achieved (7).

## 2. Objectives

In our multicenter study, the changes in sperm parameters of patients with only unidentified male infertility who received ubiquinol (QH2), reduced for of coenzyme Q10 (Co-Q10), for six months were retrospectively evaluated.

## 3. Patients and Methods

In this retrospective study, we evaluated 62 males married for at least one year who were admitted for infertility treatment without very low sperm count, and had received 100 mg QH2 twice a day for six months since January 2012 to January 2013. The participants were recruited by investigating the database of the outpatient general urology, andrology, and infertility clinics in our centers. In our outpatient clinics, QH2 was still one of the empiric treatment modalities for idiopathic subfertility. In order to evaluate the association of the results in these patients with the literature, we retrospectively investigated the databases. Only infertile patients with astheno-teratozoospermia without any identified reason and with a spermatozoa concentration of higher than 13 × 10^6^/mL were included in the investigation. In this way, the study population was normospermic or mild oligospermic with ruined morphology and/or structure. For the standardization of spermiogram results, after three days of sexual abstinence, the sperm parameters of WHO criteria were evaluated (8). For the pretreatment and posttreatment spermiogram data at least two different spermiogram analysis at each visit were performed and the sperm parameters were recorded as the mean of these values. Patients whose detailed histories or physical examinations were not available, those who had varicocele or history of varicocele surgery, had systemic diseases or were under any drug treatments for the systemic diseases, were admitted with the secondary infertility, had received chemotherapy or radiotherapy, were exposed to gonadotoxic agents, had undescended testicle or history of orchiopexy, had extremely low sperm count, had azoospermia, had chromosomal anomalies, had only one testicle or history of bilateral/unilateral orchiectomy, were under antiandrogen, androgen, testosterone, aromatase inhibitors, antiestrogen, or antidepressant treatments, or had erectile dysfunction, sexual orientation defects, or decreased libido were excluded from the study. Moreover, patients with a testicle volume of lower than 12 mL under ultrasound, without at least two different spermiogram analysis for each visit, and patients whose mean sperm volumes was lower than 1.5 mL were excluded. Besides, patients whose partners had gynecological pathologies as well as operation histories or were under treatment for hormonal imbalance were excluded. Among the patients that took QH2 treatment for six months, patients who had spermiogram analysis performed according to the WHO and Kruger criteria in order to diagnose idiopathic astheno-teratozoospermia (9), with at least two spermiogram analysis for each visit, and those whose sperm concentration was not lower than normal were included in the study.

### 3.1. Statistical Analysis

Pretreatment and posttreatment data were analyzed with SPSS 14.0 (SPSS Inc., Chicago, IL, USA). Since the variables were dependent, paired sample t test was used for comparisons. The p value < 0.05 was regarded as statistically significant.

## 4. Results

In studied database, 62 patients met the inclusion criteria. The mean age of the patients was 32.4 ± 5.2 years (range, 23-50 years). The arithmetical mean of the sper miogram parameters were determined and the pretreatment mean values were as follow: concentration (10^6^/mL), 31.5 ± 24.1 (range, 13-136); percentage of fast progressive motility (*a*), 5.7 ± 6.5 (range, 0-27); percentage of *a* plus slow progressive motility (*b*), 26.5 ± 10.6 (range, 0-50); and percentage of normal morphology, 2.6 ± 1.3 (range, 0-6). After six months (24 weeks) of QH2 treatment and regarding the spermatogenesis turnover in order to increase TAC, the arithmetical mean of the spermiogram parameters were determined and the post-treatment mean values were as follows: sperm concentration (10^6^/mL), 35.4 ± 25.3 (range, 14-142); percentage of *a*, 11.5 ± 9.3 (range, 0-38); percentage of *a* plus *b*, 37.0 ± 11.9 (range, 15-67); and percentage of normal morphology, 3.1 ± 1.1 (range, 1-6).

**Figure 2.**
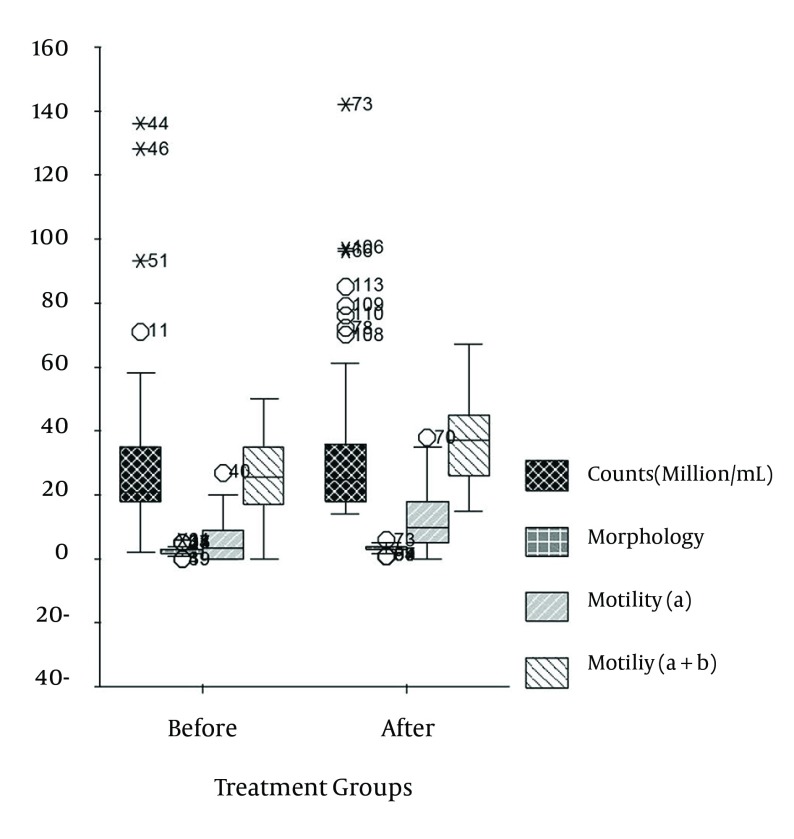
Box Plots in Pretreatment and Post-treatment Groups

Pretreatment and posttreatment sperm parameters of patients are summarized in Tables 1 and 2 as well as in Figure 2. The increase in mean values of concentration after the QH2 treatment was not statistically significant (P = 0.065). However, the changes in morphology and motility (*a* and *a* + *b*) were statistically significant (P < 0.00). Each parametric couple was compared as pair since they were dependent.

**Table 1.** Sperm Parameters of Patients in Pretreatment and Posttreatment ^a^

	Pretreatment, n = 62	Posttreatment, n = 62	P value
**Age, y**	32.4 ± 5.21	32.4 ± 5.21	
**Concentration, million/mL**	31.5 ± 24.1	35.4 ± 25.3	> 0.05
**Fast progressive, %**	5.7 ± 6.5	11.5 ± 9.3	< 0.001
**Fast + slow progressive, %**	26.5 ± 10.6	37.0 ± 11.9	< 0.001
**Normal morphology, %**	2.6 ± 1.3	3.1 ± 1.1	< 0.001

^a^ All data are presented as Mean ± SD.

**Table 2.** Sperm Parameters of Patients in Pretreatment and Posttreatment ^a^

	Pretreatment, n = 62	Posttreatment, n = 62
**Age, y**	23-50	23-50
**Concentration, million/mL**	13-136	14-142
**Fast progressive, %**	0-27	0-38
**Fast + slow progressive, %**	0-50	15-67
**Normal morphology, %**	0-6	1-6

^a^ All data are presented as minimum and maximum values.

## 5. Discussion

The bioenergetics and antioxidant activities of Co-Q10 and its reduced form, QH2, have been determined previously (10). The biosynthesis of Co-Q10 is evidently active in testicle, and the high level of QH2 in sperm plasma suggests its protective effects as an antioxidant agent (11). It has been understood that ROSs like hydrogen peroxide may cause infertility by decreasing motility and viability of spermatozoa through lipid peroxidation on the cytoplasm and membranes of spermatozoa, which are rich in very unsaturated fatty acids (12). 

There are many studies dealing with the male infertility treatment with the antioxidants such as vitamins C and E, carnitine, glutathione, selenium, and acetyl cysteine (13, 14); however, we compared our results with the few number of studies in literature concerning the effectiveness of QH2. The statistical results of our study about the empiric QH2 treatment on subfertile males with idiopathic astheno-teratozoospermia revealed that although QH2 was effective on improving sperm morphology and velocity (P < 0.00), it did not make any significant change in mean sperm concentrations in comparison with the baseline values (P > 0.05). While the pretreatment mean sperm concentrations was 31.5 ± 24.1 million/mL, the posttreatment mean sperm concentration was 35.4 ± 25.3 million/mL and the difference was not statistically significant. The pretreatment mean of normal morphology percentage was 2.6 ± 1.3 and the motility percentages were, as *a* and *a* + *b*, 5.7 ± 6.5 and 26.5 ± 10.6, respectively. After treatment, these three parameters were 3.1 ± 1.1, 11.5 ± 9.3, and 37.0 ± 11.9, respectively. The differences between pretreatment and post-treatment values of these parameters were statistically significant. Moreover, with this significant statistical improvement, the standard deviations of mean values were decreased and the range values were determined to be narrowed. However, it was revealed that the mean values of all patients did not reach the normal values according to the WHO criteria. Although the positive effects of QH2 were observed, it was clear that it could not open all the doors as a magical key in treatment of idiopathic asthenozoospermia. The results of the study by Mancini et al. (15) attracts attention; in this study, the Co-Q10 levels in seminal plasma of 77 males with normal or impaired sperm parameters were investigated and revealed that in patients with asthenozoospermia, the Co-Q10 levels in seminal plasma were significantly lower than others. This finding was the basis of our thesis and we have reported the results of empirically treated patients as a complementary of their study. It has been described that exogenous QH2 administration quickly increases semen Co-Q10 levels (16). Social security administrations are playing a determinant role not only in treatment but also in diagnosis, and more importantly, it provides evidences to declare independency of our results; we did not get any financial support. Moreover, we did not investigate Co-Q10 levels in blood or semen plasma. 

In controlled and uncontrolled trials (17, 18), it was declared that exogenous QH2 supplementation improved sperm kinetic features. However, in last three studies, the improvement in the morphology was not clearly expressed. In our study, a significant improvement in percentage of normal morphology was also determined. Considering the inverse correlation between sperm motility (fertility) and semen creatine kinase (CK), CK activities in immature sperms can be regarded as a factor of infertility (19). The investigation of CK concentrations along with morphological improvements with exogenous QH2 treatments may elucidate the physiology of these enhancements. In a more recent placebo-controlled prospective study (20), sperm parameters, Co-Q10, and hormone levels of 106 patients who received 300 mg of oral QH2 for 26 weeks (for about two spermatogenesis cycles) were investigated. Although this study was a detailed investigation, we only focused on the sperm parameters in order to compare them with our results. In this study, while the mean baseline sperm values for concentration, percent of motility (*a* + *b*), and morphology in treatment group were 20.2 ± 4.6 10^6^/mL, 27.2 ± 2.4, and 7.2 ± 2.6, respectively; the post-treatment values were determined as 26.4 ± 4.4 (P = 0.01), 27.6 ± 2.2 (P = 0.01), and 9.6 ± 2.4 (P = 0.07), respectively. In evaluation of statistical values, it was observed that not only the sperm motility but also sperm counts were increased with exogenous QH2 treatment. However, in our study we did not determine the increase in sperm count as statistically significant (P = 0.65) while morphological changes were statistically significant. In contrast to our study, the morphological changes were not statistically significant in their study (7.2 ± 2.6 and 9.6 ± 2.4 in pretreatment and posttreatment, respectively; P > 0.05). In both studies, patients with very low sperm count were excluded. While few studies evaluated these effects, there are evidences that QH2 treatment is effective mostly on motility. The subject of future study might be the determination of results of QH2 treatment on patients without any chromosomal abnormalities who have severe oligospermia. Co-Q10 is an antioxidant and bioenergetic molecule that plays a key role in mitochondrial electron transport. Fifty percent of its requirement is synthesized endogenously. Although there is not any metabolic diseases described with its deficiency in literature, Co-Q10 levels in serum or semen were not investigated in nutritional disorders such as cachexia or marasmus. Owing to its key function, in literature there are many reports concerning its practice in many medical disciplines. With respect to the sperm and its head, neck, and tail, Co-Q10 importance for urology society is its antioxidant capacity in seminal plasma and the neck region, which is rich in mitochondria producing energy necessary for the tail movements. While we shared the results of this retrospective study on empiric treatment with QH2, owing to the outcomes of previous reports, we concluded that it supported kinetic features of sperm. Moreover, we have observed that it supported the morphology. Although we determined an increase in sperm count, our results were not in agreement with the reports that claimed a significant increase in sperms count. The weakness of literature about Co-Q10 is about its effects in patients with severely diminished sperm concentration and the physiologic steps of morphologic improvements. Extended studies are warranted in order to determine the pregnancy ratios after QH2 treatment.
